# Global biogeography and evolution of *Cuvierina* pteropods

**DOI:** 10.1186/s12862-015-0310-8

**Published:** 2015-03-12

**Authors:** Alice K Burridge, Erica Goetze, Niels Raes, Jef Huisman, Katja T C A Peijnenburg

**Affiliations:** Naturalis Biodiversity Center, P.O. Box 9517, Leiden, 2300 RA The Netherlands; Institute for Biodiversity and Ecosystem Dynamics (IBED), University of Amsterdam, P.O. Box 94248, Amsterdam, 1090 GE The Netherlands; Department of Oceanography, University of Hawaii at Manoa, 1000 Pope Road, Honolulu, HI 96822 USA

**Keywords:** Zooplankton, Integrative taxonomy, Geometric morphometrics, Molecular clock, Ecological niche modelling

## Abstract

**Background:**

Shelled pteropods are planktonic gastropods that are potentially good indicators of the effects of ocean acidification. They also have high potential for the study of zooplankton evolution because they are metazoan plankton with a good fossil record. We investigated phenotypic and genetic variation in pteropods belonging to the genus *Cuvierina* in relation to their biogeographic distribution across the world’s oceans. We aimed to assess species boundaries and to reconstruct their evolutionary history.

**Results:**

We distinguished six morphotypes based on geometric morphometric analyses of shells from 926 museum and 113 fresh specimens. These morphotypes have distinct geographic distributions across the Atlantic, Pacific and Indian oceans, and belong to three major genetic clades based on COI and 28S DNA sequence data. Using a fossil-calibrated phylogeny, we estimated that these clades separated in the Late Oligocene and Early to Middle Miocene. We found evidence for ecological differentiation among all morphotypes based on ecological niche modelling with sea surface temperature, salinity and phytoplankton biomass as primary determinants. Across all analyses, we found highly congruent patterns of differentiation suggesting species level divergences between morphotypes. However, we also found distinct morphotypes (e.g. in the Atlantic Ocean) that were ecologically, but not genetically differentiated.

**Conclusions:**

Given the distinct ecological and phenotypic specializations found among both described and undescribed *Cuvierina* taxa, they may not respond equally to future ocean changes and may not be equally sensitive to ocean acidification. Our findings support the view that ecological differentiation may be an important driving force in the speciation of zooplankton.

**Electronic supplementary material:**

The online version of this article (doi:10.1186/s12862-015-0310-8) contains supplementary material, which is available to authorized users.

## Background

Shelled pteropods (Mollusca, Gastropoda: Thecosomata) are potentially good bioindicators of the effects of ocean acidification, but their application as such is hampered by limited knowledge of their taxonomy, genetic diversity, ecology and distribution patterns. Pteropods are a group of heterobranch gastropods [1] that are a common component of the marine zooplankton. They affect the ocean carbon cycle by producing aragonite shells that can accelerate the export of organic matter from the surface into the deep ocean. Because of their delicate aragonite shells, pteropods have been identified as exceptionally vulnerable to rising CO_2_ (e.g. [2-4]), and hence are widely used to explore the effects of ocean acidification (e.g. [5-8]). Shelled pteropods also may be a particularly informative model system for the study of long-term marine evolutionary processes, because they are metazoan plankton with an abundant fossil record (e.g. [9,10]).

Recent studies suggest that marine plankton have higher evolutionary potential than originally thought and may be well poised for evolutionary responses to global change (e.g. [11-13]). A recent review of population genetic studies of oceanic zooplankton showed that genetic isolation can be achieved at the scale of gyre systems, and appears to be linked to the particular ecological requirements of the organisms [14]. Molecular phylogenetic studies on calcifying plankton have suggested greater specificity in oceanographic habitat preferences than previously supposed, e.g., in coccolithophores (e.g. [15,16]) and foraminifers (e.g. [16-22]). The evolutionary potential of calcifying plankton is further supported by long-term selection experiments, which have demonstrated rapid functional genetic divergence in response to elevated CO_2_ concentrations [11,12]. Hence, calcifying plankton may be capable of rapid evolutionary as well as ecological responses to changing ocean conditions, including future changes driven by global warming and ocean acidification.

The taxonomy of shelled pteropods has generally been based on shell morphology, although some studies have also examined soft parts (e.g. [23,24]). Several pteropod taxa have been identified using the traditional approach of univariate measurements of shell dimensions (e.g. [24-27]). Yet, the complex and highly diverse shell morphologies of pteropods also enable detailed geometric morphometric analyses. Several studies have shown that geometric morphometrics can be more powerful in distinguishing taxa than univariate measurements (e.g. [28-30]). Molecular phylogenetic studies suggested that taxonomic revisions will be required [31-33]. These studies showed a well-supported separation of species and genera, but relationships among genera are poorly resolved. The first genus-level study of pteropods focused on DNA barcoding of *Diacavolinia*, and emphasized the inadequacy of current systematic understanding of this genus [34]. The taxonomy of *Creseis*, *Hyalocylis* and *Styliola* was reviewed by [35].

This study focuses on the genus *Cuvierina*, an excellent model group for an integrative study of zooplankton because it has a worldwide distribution, it is abundantly present in museum collections, and has a well-described fossil record [25]. Moreover, the bottle-shaped adult shells (6–11 mm in length) do not change during adult life, and can be easily distinguished from juvenile shells that are shed once the animal is mature [10,36]. Extant *Cuvierina* pteropods occur from 45°N to 40°S, in ocean regions with surface water temperatures above ~17°C [23]. Recent taxa are absent from the Mediterranean and Red Seas [25,37]. *Cuvierina* taxa have a diel vertical migration pattern and prefer epipelagic depths, with highest abundance between 100 and 250 m [37]. *Cuvierina* taxa are hermaphrodites and internal fertilizers [10]. The most recent taxonomic revision of *Cuvierina* was based on univariate shell measurements and a description of shell micro-ornamentation [25,38]. Based on fossil evidence [25] proposed that extant *Cuvierina* species evolved 5–4 million years ago (mya), with the origin of the first fossil species estimated at 25–24 mya. Janssen [25] proposed a subdivision of five extant morphospecies divided in two subgenera: *Cuvierina (Urceolarica) cancapae* [25], *Cuvierina (Urceolarica) urceolaris* [39], *Cuvierina (Cuvierina) columnella* [40], *Cuvierina (Cuvierina) atlantica* [36], and *Cuvierina (Cuvierina) pacifica* [25]. However, recent studies of pteropods have not implemented this species-level revision, resulting in considerable taxonomic confusion [31-33,41]. Here, we follow and test the morphological taxonomy of [25], referring to the proposed *Cuvierina* taxa as morphotypes.

The overall aim of this study was to obtain a framework of phenotypic, genetic and geographic information to assess species boundaries and the evolutionary history of *Cuvierina* pteropods. To this end, we first applied geometric morphometric analyses of shell outlines using an extensive collection of museum and fresh specimens from the Pacific, Atlantic, and Indian Oceans. Secondly, we sequenced a portion of mitochondrial DNA (mtDNA) comprising a fragment of the cytochrome oxidase I (COI) subunit, as well as a portion of the nuclear 28S rDNA gene. Thirdly, we plotted global distribution patterns of *Cuvierina* morphotypes and applied ecological niche modelling to estimate their ecological tolerances. Our specific objectives were (1) to distinguish between and within extant taxa using an integrative approach (as suggested by e.g. [42]), (2) to determine the temporal sequence of evolution in the genus using a fossil-calibrated molecular phylogeny, and (3) to explore the current and past biogeographic context of extant *Cuvierina* taxa.

## Methods

### Samples

**Figure 1:**
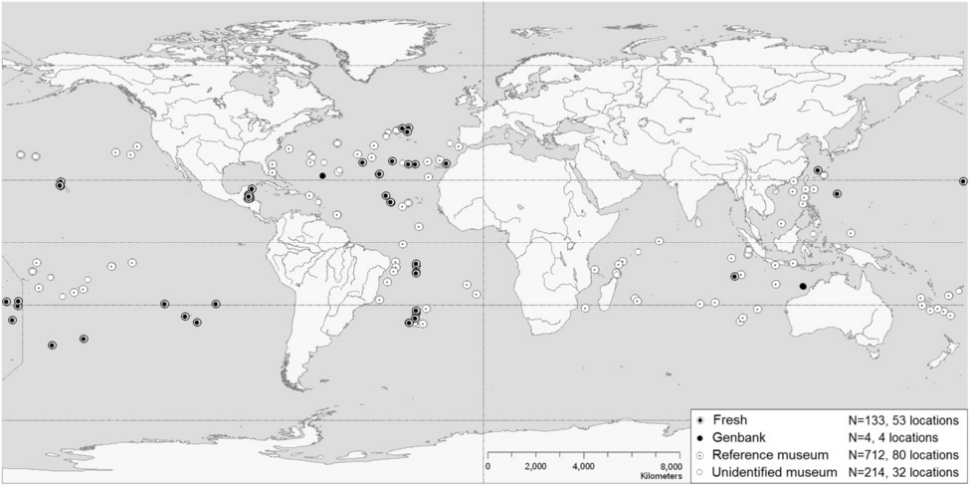
**Geographic overview of all** ***Cuvierina*** **specimens used in this study.** Some sampling locations of reference and unidentified museum samples overlap.

An overview of all *Cuvierina* samples is listed in Table 1 (more detailed information can be found in Additional file 1). Museum samples included specimens from the Natural History Museum of Denmark in Copenhagen (ZMUC) and Naturalis Biodiversity Center (NBC, Leiden, formerly Zoological Museum of Amsterdam (ZMA)). Museum samples were stored in 70% ethanol, but all museum samples had initially been fixed in formalin rendering them unsuitable for genetic analyses. Geographic locations of museum samples were either provided with the samples or obtained from [43-46] or [47]. Samples from ZMUC (N = 712 from 80 locations, 1–46 specimens per sample) were identified and sorted per morphospecies by A.W. Janssen (2001–2005 [25]) and served as reference museum samples for geometric morphometric analyses in this study (Figure 1, Table 1 and Additional file 1). Reference samples were collected during the Danish DANA expeditions between 1911 and 1934 during all seasons (53 locations), and during various other expeditions between 1846 and 1912 (27 locations) such as Leg. Andrea (Additional file 1: Table S1). Samples from NBC (N = 214 from 32 locations, 1–23 specimens per sample) were not identified according to the taxonomic revision of *Cuvierina* by [25] and are referred to as unidentified museum samples (Figure 1, Table 1). These samples were collected during the DANA expeditions between 1911 and 1933 (21 locations), ACRE expeditions (1967–1968, 5 locations), and Project 101A (1980, 6 locations) (Additional file 1). Fresh samples (N = 133 from 53 locations, 1–23 specimens per sample) were collected between 2001 and 2012 during the following expeditions: MP3 (2001, 3 locations), 0106TRAN (2001, 1 location), COOK 11MV and 14MV (2001, 5 locations), DRFT07RR (2001, 3 locations), VANC10MV (2003, 1 location), MARECO (2004, 3 locations), ECO-CH-Z (2007, 9 locations, provided by R.A. Gasca Serrano, Unidad Chetumal, Mexico), AMT18 (2008, 3 locations), R/V Tansei-Maru KT-10-20 (2010, 1 location, provided by H. Miyamoto, University of Tokyo, Japan), Kilo Moana 1109 (2011, 4 locations), AMT22 (2012, 14 locations), and KH-11-10 (2011–2012, 6 locations, provided by A. Tsuda, University of Tokyo, Japan) (Figure 1, Additional file 1). The collection nets used had mesh sizes between 0.2 and 1 cm. Fresh samples were stored in 96% ethanol. Only mature individuals with intact shells, both museum and fresh specimens, were used for geometric morphometric analyses. All fresh individuals were used for genetic analyses (N = 133) (Table 1 and Additional file 1).

**Table 1:** **Overview of** ***Cuvierina*** **samples used in this study**

**Morphotype**	**Ocean**	**Morphometrics ventral**	**Morphometrics apertural**	**COI**	**28S**
	**Total**	**1039**	**550**	**136**	**31**
**Reference museum samples**		**712**	**352**		
***C. atlantica***		**226**	**83**		
	North Atlantic	218	75		
	South Atlantic	8	8		
***C. cancapae***		**103**	**43**		
	Central Atlantic	103	43		
***C. columnella***		**65**	**39**		
	Indian	30	20		
	Pacific	35	19		
***C. urceolaris***		**226**	**95**		
	Indian	137	49		
	Pacific	89	46		
***C. pacifica*** **N**		**34**	**34**		
	Pacific	34	34		
***C. pacifica*** **S**		**58**	**58**		
	South Pacific	58	58		
		**58**	**58**		
**Unidentified museum samples**		**214**	**83**		
	Atlantic	168	37		
	Pacific	33	33		
	Indian	13	13		
**Unidentified fresh samples**		**113**	**115**	**133**	**31**
	Atlantic	60	61	72	14
	Pacific	52	53	60	15
	Indian	1	1	1	1
Genbank sequences*				**3**	**1**
	Atlantic			1	1
	Indian			2	0

*GenBank sequences: COI, Atlantic: FJ876895, Indian: FJ876896–7 [32]; 28S, Atlantic: DQ237984 [33].

### Geometric morphometrics

**Figure 2:**
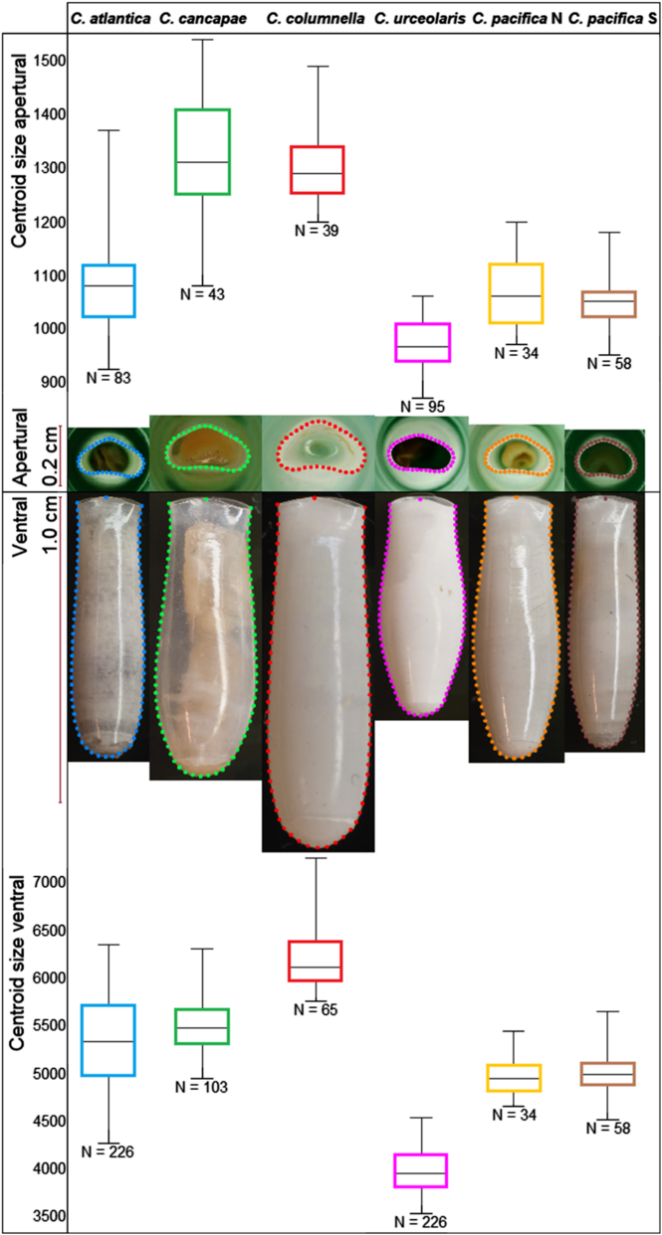
**Centroid size variation for reference museum specimens of** ***Cuvierina*** **in ventral (N = 712) and apertural (N = 352) orientations.** Typical specimens of six morphotypes are shown with outlines transformed to semi-landmarks to allow for geometric morphometric analysis. Lengths of *Cuvierina* shells were between 0.6 and 1.1 cm.

Specimens were photographed in two orientations: ventral and apertural (Figure 2). Photographs were taken with a Nikon D100 6 mpx camera (Micro-Nikkor lens 55 mm/3.5, aperture f/16, shutter speed 1/8 s ISO 200, fixed zoom), which was attached to a stand. For ventral photography, specimens were mounted on photographic film with methyl glucose (60%) to standardize the orientation. For apertural photography, shells were put into small disposable pipette tips. Photographs were adjusted in Adobe Photoshop Lightroom 2–2008 on white balance, sharpness, vibrancy and noise. Files containing pictures selected for analysis were prepared using tpsUtil [48]. Only well-focused, undamaged adult shells in standardized orientation were used.

Because there are no true landmarks on *Cuvierina* shells, we used semi-landmarks for outlining shells [49]. Ventral shell outlines were created in tpsDig [48] for a total of 1039 specimens, and apertural outlines were applied to a subset of 550 specimens (Figure 2, Table 1). The ventral outline was created by starting and ending at the distinct transitions from the outside of the shell to the aperture. One separate semi-landmark was placed at the top of the aperture. Using tpsUtil [48] the outline was converted to 75 semi-landmarks, separated by equal length, enabling further analyses (Figure 2). The apertural outline started with the semi-landmark in the middle of the aperture edge (the upper semi-landmark shown for typical specimens in Figure 2), being one of 35 semi-landmarks in which the apertural outline was converted. TpsRelw [48] was used to rotate, translate and scale semi-landmark coordinates through generalized least square Procrustes superimposition (GLS) ([50], in [51]). GLS provided centroid sizes (a size measure depending on surface area) and multiple relative warp axes (RWs; ventral N = 148; apertural N = 70) per specimen. RWs contain information on shape, with the first RW containing the most shape information. To test for repeatability of RWs, a selection of 44 specimens was photographed in two subsequent series. Intraclass correlation coefficients (ICCs) between the two series were calculated for the first 10 RWs in Past 3.0 [52]. RWs were considered repeatable when ICC > 0.80. The outline method for geometric morphometric analyses on *Cuvierina* shells was highly repeatable (ICC > 0.89 for ventral RWs 1, 2, 4, 5, 6 and centroid size; ICC > 0.82 for apertural RWs 1, 2, 4, 5 and centroid size). Only repeatable RWs were used in further analyses of shell shape. To test whether sliding semi-landmarks provided more consistent results, ICCs were also calculated after transformation of semi-landmarks separated by equal-length intervals into sliding semi-landmarks with estimated positions on the outline. This did not improve repeatability; hence only semi-landmarks were used in this study.

The *a priori* classification of reference museum specimens (those identified by [25]), was compared to results of clustering by linear discriminant analyses (LDA) in R 3.0.1 [53]. Parameters for identifying morphotypes using LDA were ventral centroid size and ventral RWs 1, 2, 4, 5, 6. Subsequently, we compared our manual morphotype identifications of previously unidentified museum and fresh specimens to results of LDA-assignment of these specimens to morphotypes. Our manual identification of unidentified specimens was based on ventral centroid size, ventral RWs 1, 2, 4, 5, 6, and if available, apertural centroid size and apertural RWs 1, 2, 4, 5. The performance of the LDA algorithm was tested by cross-validation using a jackknifed confusion matrix in Past 2.17c [52].

To test for significant differentiation between *a priori* defined groups of *Cuvierina* (morphotypes or geographic distributions), we applied non-parametric permutational multivariate analyses of variance (PerMANOVA, [54]) in Past 2.17c. We used Euclidean distances applied to ventral and apertural centroid sizes and RWs with ICC > 0.80 (representing 96.75% of shell shape variation). The PerMANOVA *F*-statistic was tested against 9999 nonparametric permutations.

### Genetics

To assess levels of genetic variation within *Cuvierina*, we sequenced 133 individuals for COI mtDNA and 31 individuals for 28S rDNA (Table 1). Entire juveniles (N = 4) and 2 x 2 mm of tissue of adults (N = 129) were individually placed in BLB buffer (250 mM EDTA, 5% SDS, 50 mM Tris–HCl pH 8.0; [55]) for at least 24 h. Total DNA was extracted from BLB buffer using the DNeasy blood & tissue kit (Qiagen Benelux B.V. 2006). The extract was resuspended in 100 μl AE-buffer (Qiagen Benelux B.V.). A 658 bp fragment of COI was amplified using the primers LCO1490 (5′-GGTCAACAAATCATAAAGATATTGG-3′) and HCO2198 (5′-TAAACTTCAGGGTGACCAAAAAATCA-3′) [56]. For a subset of 31 individuals, a 965 bp 28S rDNA fragment was amplified using the primers 28SC1F (5′-ACCCGCTGAATTTAAGCAT-3′) and 28SD3R (5′-GACGATCGATTTGCACGTCA-3′) [57]. Most polymerase chain reactions (PCR) of COI and 28S, with total volumes of 25 μl, contained PCR Beads (GE Healthcare Europe GmbH) with 3 μl template DNA, 0.5 μl of each primer, and 21 μl ddH_2_O. Alternatively for COI, PCR solutions (25 μl) contained 3 μl template DNA, 2.5 μl 10x reaction buffer (HT Biotechnology, Cambridge, U.K.), 1 μl MgCl_2_ (25 mM), 2.5 μl dNTPs (GATC 1 mM each), 0.3 μl of each primer (10 μM), 0.15 μl Taq polymerase (HT Biotechnology), 0.2 μl BSA (10 mg/ml) and 15.05 μl ddH_2_O. Amplifications were carried out in a PTC-200 DNA Engine Cycler (Bio-Rad Laboratories B.V.) with an initial denaturation of 3 min at 94°C, 35 cycles of 45 s at 94°C, 1 min at 45°C, and 1.5 min at 72°C, followed by final extensions of 10 min at 72°C and 5 min at 4°C. Sequencing of PCR products was carried out using both PCR primers by Macrogen Europe. The genus-level identities of all sequences were confirmed by BLAST searching GenBank [58]. Sequences were aligned in CodonCode Aligner 4.1 (CodonCode Corporation, USA, 2013). Additional GenBank sequences were added, namely three for COI (‘*C. columnella*’): FJ876895–7 [32] and one for 28S (‘*C. columnella*’): DQ237984 [33].

To estimate evolutionary relationships among COI haplotypes, we applied a Maximum Likelihood (ML) approach [59] in raxmlGUI 1.3, which only provides GTR-related models of rate heterogeneity for nucleotide data [60,61]. We tested for the most appropriate model of sequence evolution for this dataset using AIC in jModelTest 2.1.3 [62] based on 88 models and the GTR + Γ + I was selected. However, we use 3 codon positions (CP) instead of Γ + I because it is a biologically realistic model for protein coding sequences (following [63]). We applied a ML search followed by a non-parametric bootstrap analysis with 2000 replicates. Additionally, we calculated pairwise genetic distances for COI in MEGA 6.0 using the p-distance model of evolution [64].

To test for congruence of mitochondrial clades with nuclear DNA, we reconstructed alleles from 28S genotypes using the PHASE algorithm [65,66] in DnaSP v5 [67] and used these to calculate pairwise genetic distances with the p-distance model of evolution in MEGA 6.0.

We further explored the population genetic structure of *Cuvierina* in the Atlantic Ocean based on the COI fragment using a total of 60 fresh specimens for which morphotypes were assigned (northern *C. atlantica* (N = 34), southern *C. atlantica* (N = 21) and central Atlantic *C. cancapae* (N = 5); Additional file 1). The number of fresh specimens from the Indian and Pacific Oceans was insufficient for population genetic analyses. We obtained haplotype diversity (H) and nucleotide diversity (π, [68,69]) for each population sample and pooled samples per morphotype and/or geographic region using Arlequin 3.5.1.3 [70]. We tested for differentiation between Atlantic morphotypes and between geographic regions within morphotypes (e.g. North Atlantic versus South Atlantic) using φ_ST_ based on pairwise differences. These were tested for divergence from the null distribution of no differentiation with 10 000 permutations, as implemented in Arlequin. For all analyses involving multiple simultaneous tests, significance levels were adjusted by application of a sequential Bonferroni correction with an initial alpha of 0.05 [71].

To reconstruct evolution within *Cuvierina* and to provide a phylogenetic perspective of outgroup relationships, 30 *Cuvierina* sequences were compared to other Cavoliniid taxa. This approach was applied to combined partitions of COI (658 bp) and 28S (989 bp). Outgroup taxa were *Creseis conica*, *Clio pyramidata*, *C. cuspidata*, *C. recurva*, *Diacria danae* and *D. trispinosa*. The AIC in jModelTest 2.1.3 based on 88 models suggested the use of GTR + Γ + I for COI and GTR + Γ for 28S. Firstly, we applied a ML search followed by non-parametric bootstrap analysis with 3500 replicates in raxmlGUI 1.3. For this purpose, we used the GTR + CP substitution model for COI following [63]. Secondly, we applied a relaxed Bayesian molecular clock analysis to combined COI and 28S partitions with uncorrelated lognormal rates in BEAUti and BEAST 1.7.5 [72]. For this we used the models suggested by jModelTest 2.13, because CP-based reconstructions failed to reach an Effective sample size (ESS) > 100 for the posterior statistic after two runs of 10^9^ generations (burn-in 2 × 10^8^ generations), as visualized in Tracer 1.5 [73]. The tree prior was set to the Yule Process of speciation [74] with a random starting tree. Because our dataset consists of intraspecific as well as interspecific sequences, we limited our dataset to one individual per taxon, but used two individuals of *C. pacifica* S to calculate the TMRCA of this clade. We included the most basally positioned individuals for each morphotype based on the ML-phylogeny of COI and 28S combined. Two fossil calibrations were used, one on the root node of the tree (= stem Cavolinoidea) and one for the time of most recent common ancestry (TMRCA) of extant *Cuvierina*. For the first calibration we used the first fossil occurrence of the now extinct Cavoliniid *Camptoceratops priscus* [75], 47.8–56 mya (Ypresian stage, A.W. Janssen, pers. comm. and in accordance with [31]) and set a lognormal distributed prior (log (Mean) = 8.0; log (Stdev) = 0.7; offset = 48.0). For the crown node of *Cuvierina* we set a lognormal distributed prior (log (Mean) = 3.0; log (Stdev) = 0.5; offset = 23.0) based on the first occurrence of *Cuvierina torpedo* in the fossil record at 20.4–23 mya [25,76]. The preliminary MCMC chain was 10^7^ generations (burn-in 10^6^ generations), followed by six runs of 2.5 × 10^8^ generations (burn-in 2.5 × 10^6^ generations each). We sampled trees and log-likelihood values at 10 000-generation intervals. Sets of trees obtained during independent runs were combined in LogCombiner 1.7.5 [72] and the maximum clade credibility tree was selected using TreeAnnotator 1.7.5 [72].

### Ecological niche modelling

To test whether *Cuvierina* morphotypes were ecologically differentiated based on their biogeographic distributions, we applied ecological niche modelling (ENM) to estimate their ecological tolerances. Based on geometric morphometric analyses, we plotted global morphotype occurrences on maps containing marine environmental data from the Bio-ORACLE dataset [77] in a WGS1984 coordinate system in ArcMap 10.0 (ESRI LTD., USA, 2011) to obtain an indication of geographic distributions in relation to the ecological variables. The number of georeferenced sampling locations for each morphotype was 69 for *C. atlantica*, 14 for *C. cancapae*, 17 for *C. columnella*, 30 for *C. urceolaris*, 20 for *C. pacifica* N, and 20 for *C. pacifica* S, respectively. We calculated Spearman rank correlation coefficients (ρ) between the environmental variables and performed a principal component analysis (PCA) on all Bio-ORACLE data layers. The PCA was used to select the most informative ecological variables from sets of correlated variables. This reduced the effect of collinearity of environmental variables [78]. Six uncorrelated data layers (with ρ ranging from-0.48 to 0.72), all with a spatial resolution of 5 arcmin (*ca.* 9.2 km), were selected for ENM: maximum monthly sea surface temperature (SST), annual SST range, annual average sea surface salinity (SSS), annual average surface pH, maximum monthly photosynthetically active radiation reaching the ocean surface (PAR) and maximum monthly near-surface chlorophyll *a* concentration. The Bio-ORACLE layers SST, chlorophyll *a* and PAR were based on remotely sensed data [77]. Maximum monthly chlorophyll *a* concentration was set to a maximum of 10 mg/m^3^ and annual average SSS was set to a minimum of 30 PSU as seen in nature. Although *Cuvierina* taxa are most abundant between 100 and 250 m, they migrate daily between surface waters and greater depths [37]. It is therefore likely that sea surface variables are an important dimension of *Cuvierina*’s niche. Using MaxEnt 3.3.3 k [22,79] we created response curves for these six ecological variables, performed jackknife tests to measure the importance of individual environmental variables in explaining the modelled distribution of each morphotype, and estimated potential niches per morphotype. Response curves were not extrapolated outside the range of observed values. We used the default settings and all presence records (N = 170) for training our model. Accuracy of ENMs per morphotype was examined using a null-model methodology using 99 randomisations that allows for significance testing of ENMs [80]. This test corrects for collection bias by restricting the randomly drawn points to all known sampling locations (presence-only data). Niche overlap between pairs of morphotypes was calculated by the Schoener’s *D* statistic [81,82] in ENMTools [83]. A *D*-value of 1 indicates that two species share the same environmental space and a *D*-value of 0 suggests no overlap.

## Results

### Phenotypic variation

**Figure 3:**
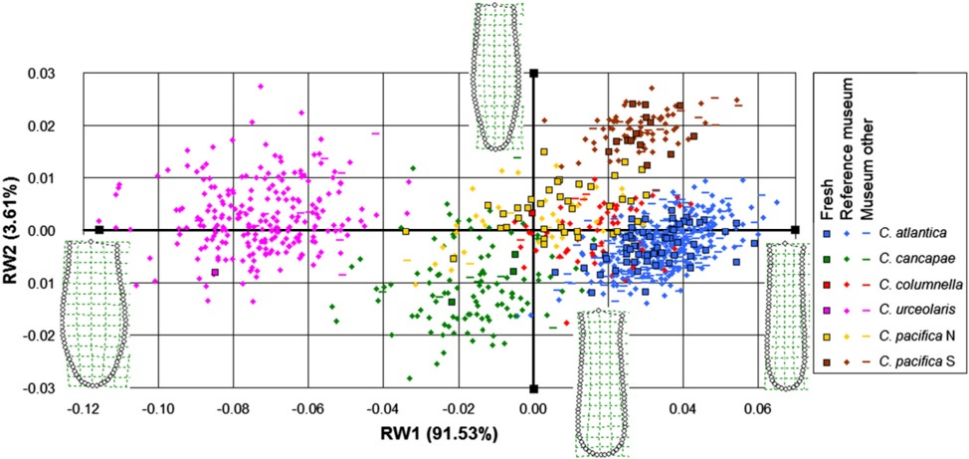
**Ordination of uncorrected Relative Warp (RW) data of** ***Cuvierina*** **in a ventral orientation.** Fresh (N = 113), reference museum (N = 712) and other museum specimens (N = 214) are included. Relative Warp 1 explains 91.53% of the total shape variation; RW2 explains 3.61%. Corresponding thin plate splines of the most positive and negative deformations along the axes are indicated to depict the variation in shell shape. Six distinguished morphotypes are indicated in the legend.

We distinguished six morphotypes in the pteropod genus *Cuvierina* based on geometric morphometric analyses of shell shape and size of reference specimens (Figures 2 and 3). Because we found a separation between the North and South *C. pacifica* morphotypes (Figures 2, 3 and Additional file 2, respectively), these were separated in further analyses and are referred to as *C. pacifica* N and *C. pacifica* S. All morphotypes were significantly different from each other in terms of centroid size (ventral *F* = 731.7; *p* < 0.001) except *C. pacifica* N and *C. pacifica* S (Figure 2). Separation in size and shape was most evident in ventral orientation. Here, the overall shell shape variation between six morphotypes was significant after strict Bonferroni correction (N = 712, 96.89% of shape variation; *F* = 1364; *p* < 0.001). Extremes on the first RW axis (explaining 91.53% of ventral shell shape variation) were represented by the cylindrical *C. atlantica* and the bottle-shaped *C. urceolaris*, respectively (Figure 3). Extremes on the second RW axis (explaining 3.61% of ventral shell shape variation) were *C. pacifica* S with a narrow shell bottom (septum) and *C. cancapae* with a broad septum, respectively. In an apertural orientation, two of the six morphotypes (*C. urceolaris* and *C. pacifica* S) were significantly differentiated (N = 352; 84.59% of shape variation; *F* = 232.1; *p* < 0.001).

We also found significant variation in shell shape and size *within* morphotypes for *C. columnella* and *C. urceolaris* from different geographic regions. *C. columnella* specimens from the Indian Ocean (N = 30) were significantly larger than Pacific specimens (N = 35; *F* = 25.44; *p* < 0.001). The opposite was true for *C. urceolaris*: specimens from the Indian Ocean (N = 137) were significantly smaller than Pacific specimens (N = 89; *F* = 123.7; *p* < 0.001). We found no significant shape and size differences between *C. atlantica* specimens from the northern (N = 255) and southern (N = 28) Atlantic populations.

Linear Discriminant Analyses (LDA) almost completely matched manual morphotype identifications based on geometric morphometric analyses of unidentified specimens and showed high correspondence with *a priori* classification of reference museum samples. Without assigning samples to ocean basins *a priori*, 96% of all reference specimens (N = 712) were assigned to the same morphotype as determined *a priori*. Confidence increased to 99% when samples from the Atlantic and Indo-Pacific were analysed separately. We compared this assignment method to LDA and found that without separating samples from the Atlantic and Indo-Pacific basins, 91% of all fresh specimens (N = 113) and 96% of all unidentified museum specimens (N = 214) were assigned to the same morphotypes as in manual identifications. This accuracy increased when samples were distinguished geographically: 96% of all fresh specimens and 99.5% of unidentified museum specimens were assigned to the same morphotype as with our manual method. We chose our manual method as the final identification method because a few specimens could not be identified unambiguously by LDA. These specimens had either shapes or centroid sizes that were on the edges of the total size-range or morphospace of a specific morphotype. Cross-validation demonstrated a high accuracy of the LDA algorithm itself: 98% of Atlantic and 99.5% of Indo-Pacific reference museum samples were identified as true positives.

### Genetic variation

**Figure 4:**
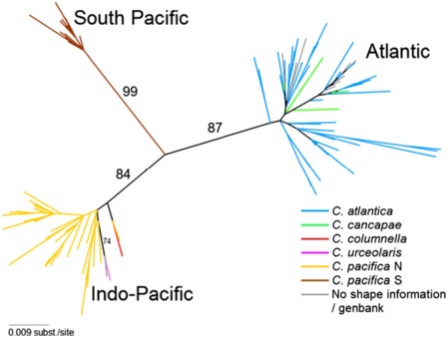
**Unrooted maximum likelihood tree of 136 Cytochrome Oxidase I gene sequences of** ***Cuvierina*** **.** Three sequences are from GenBank: FJ876895, Atlantic; FJ876896-7, Indian Ocean. Numbers indicate bootstrap support (only bootstrap values of major clades are shown). Symbols indicate major genetic clades; colours indicate distinct morphotypes (also see Figure 5).

**Figure 5:**
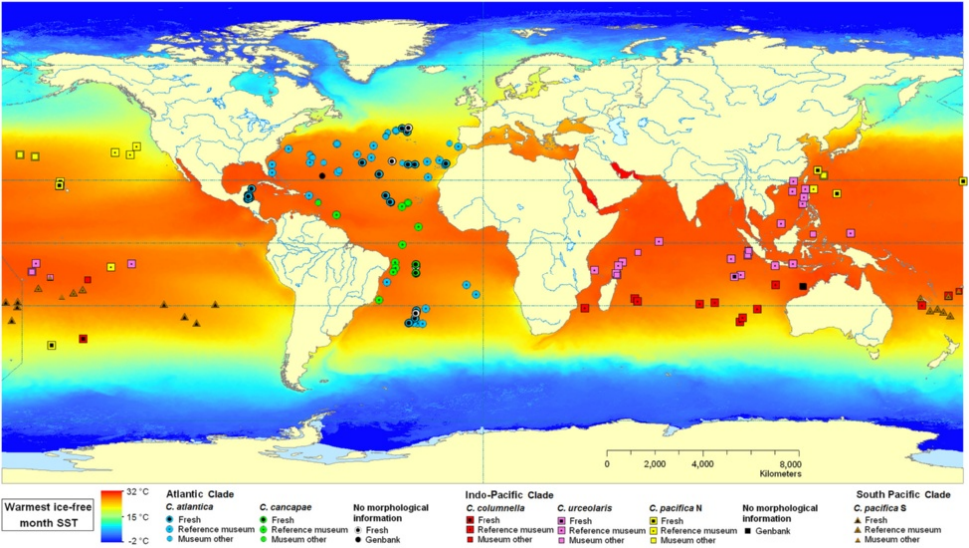
**Geographic overview of all** ***Cuvierina*** **specimens used in this study.** Sampling locations are projected on a map of sea surface temperatures (SST) of the warmest ice-free month (MARSPEC data set, [84]). See legend for explanation of symbols and colours.

We found high levels of mitochondrial diversity in a data set of 136 COI sequences collected from global samples of *Cuvierina*, including 127 different haplotypes represented by 166 polymorphic sites (GenBank accession numbers KP292656–KP292788; Additional file 1). We translated COI sequences into amino acids and discarded the possibility of pseudogenes because we found no stop codons and no insertions or deletions. Phylogenetic analysis of COI sequences indicated the presence of three major mitochondrial clades (Figure 4 and Additional file 3). These three monophyletic clades were highly supported (bootstrap values of 84–99%) are largely congruent with morphotypes as well as geographic distributions (Figure 5). The three major clades were named after their geographic distributions; viz., Atlantic, Indo-Pacific and South Pacific (Figures 4 and 5). The Atlantic clade contains both the *C. atlantica* and *C. cancapae* morphotypes: we did not find any grouping of these morphotypes, nor did we find any grouping of individuals from either the North or South Atlantic. The Indo-Pacific clade consists of *C. urceolaris*, *C. pacifica* N and *C. columnella* morphotypes. Within this clade, *C. pacifica* N was paraphyletic. Our single specimen of the *C. urceolaris* morphotype grouped with two GenBank sequences from the Indian Ocean (both reported as *C. columnella*, [32]). The South Pacific clade consists entirely of the *C. pacifica* S morphotype. Average pairwise genetic distances of COI were 4.5–5.1% between major clades and 2.0%, 1.7% and 0.8% within clades for the Atlantic, Indo-Pacific and South Pacific, respectively (Additional file 4).

Genetic patterns in 28S were not in conflict with our COI data but contained much less variation (0.8% for N = 31 representing 6 morphotypes). We found 11 diploid genotypes represented by 13 phased alleles and a total of 8 polymorphic sites (GenBank accession numbers KP292620–KP292649; Additional files 1 and 4). Most *Cuvierina* morphotypes shared 28S sequences, however, *C. pacifica* S and our single *C. urceolaris* specimen had unique single substitutions at positions 866 and 678, respectively (both C instead of T). Average pairwise genetic distances of 28S were 0.03%, 0.1% and 0.09% within the Atlantic, Indo-Pacific and South Pacific mitochondrial clades, and 0.14–0.26% between major clades (Additional file 4).

Because we found *C. atlantica* populations in the northern as well as southern Atlantic Ocean, which are separated geographically by *C. cancapae* (Figure 5), we tested for spatial genetic structuring. Overall, we found high levels of genetic diversity in Atlantic *Cuvierina* samples (haplotype diversities ranged from 0.99 to 1.0 per sample). Nucleotide diversities were comparable for northern *C. atlantica* (π = 0.020 ± 0.01, N = 34), southern *C. atlantica* (π = 0.022 ± 0.01, N = 21) and *C. cancapae* (π = 0.020 ± 0.01, N = 5). We found significant population genetic structuring of northern versus southern *C. atlantica* populations (φ_ST_ = 0.047, *p* = 0.008), but not between *C. cancapae* and any *C. atlantica* population. This could be due to low sample size of *C. cancapae*.

**Figure 6:**
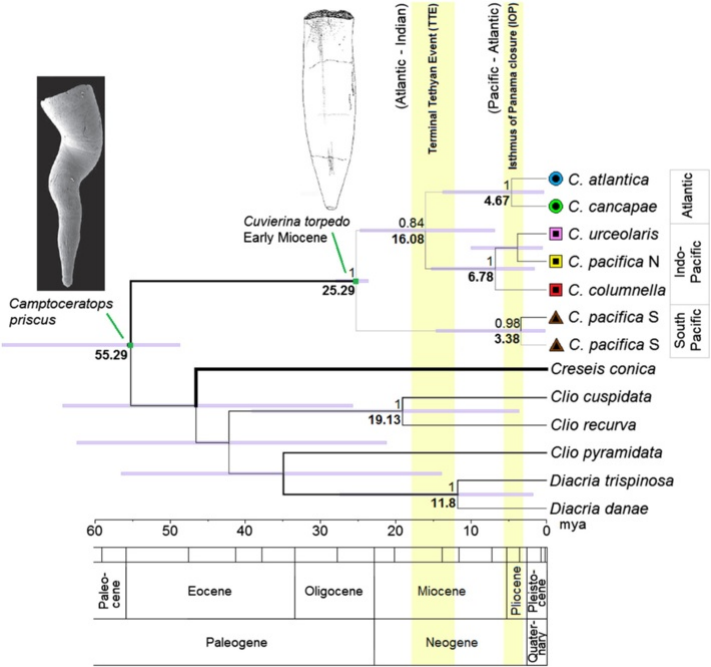
**Fossil calibrated Bayesian phylogeny of 7** ***Cuvierina*** **and 6 outgroup taxa using COI (658 bp) and 28S (989 bp).** The reconstruction was calibrated by the earliest occurrences in the fossil record of *Camptoceratops priscus* and *Cuvierina torpedo*, indicated by green dots on the nodes. Numbers above major branches indicate posterior probabilities (only values >0.95 are shown); numbers below major branches indicate ages in million years ago. Branch widths correspond to substitution rates, with thick branches indicating high substitution rates. Symbols for *Cuvierina* indicate major genetic clades; colours indicate morphotypes (also see Figure 5). Image of *C. priscus*: [85]; and *C. torpedo*: [76]. The TTE and IOP are highlighted after estimations by [86].

We reconstructed evolution within the genus *Cuvierina* based on ML and fossil-calibrated Bayesian phylogenetic analyses of the combined COI + 28S sequence data (Additional file 3 and Figure 6, respectively). The ML and Bayesian reconstructions established a well-supported monophyly of *Cuvierina* versus outgroup taxa (GenBank accession numbers KP292650–KP292655 and KP292789–KP292794; Additional file 1). Within *Cuvierina*, the South Pacific clade appears basal in both reconstructions, followed by a split between the Atlantic and Indo-Pacific. We found that evolutionary rates of COI and 28S in outgroup taxa were highly variable and outgroup relationships remained largely unresolved (PP < 0.50; ML bootstraps < 40). *Creseis*, the sister taxon of *Cuvierina* based on interpretation of fossil evidence by [25], had the fastest evolutionary rate with respect to the other taxa based on our Bayesian analyses (Figure 6). Following the TMRCA of 25.3 (28–23, 95% confidence intervals) mya (Oligocene) for the genus *Cuvierina*, the first divergence most likely took place between the South Pacific and the Indo-Pacific/Atlantic clades. The Indo-Pacific and Atlantic clades diverged ~16.1 (24.5–7) mya (Miocene). The TMRCA for recent taxa within these three major clades was estimated at least 4.7 (13.5–1) mya for the Atlantic, 6.8 (15–2) mya for the Indo-Pacific, and 3.4 (14.5–0.5) mya for the South Pacific clades.

### Biogeography

*Cuvierina* morphotypes are restricted to warm tropical and subtropical oceanic waters between ca. 43° north and ca. 40° south (Figure 5). We found little range overlap between *Cuvierina* morphotypes, especially in the Atlantic and Indian Oceans. However, Pacific distributions of morphotypes of the Indo-Pacific clade (*C. columnella*, *C. pacifica* N and *C. urceolaris*) partly overlapped (Figure 5). In the Atlantic Ocean, the *C. cancapae* morphotype is found in equatorial waters, extending as far as 23° south, whereas *C. atlantica* appears in subtropical waters up to 43° north and 35° south. In the Indian Ocean, *C. urceolaris* is found in equatorial waters, whereas *C. columnella* appears in subtropical waters up to 35° south. In the Pacific Ocean, *C. urceolaris* is found in equatorial waters, but *C. columnella* appears in equatorial as well as southern waters up to 40° south. The *C. pacifica* N morphotype has a much wider distribution pattern. It is confined to the Pacific Ocean and has been observed in northern subtropical waters up to 38° north, but also at a few equatorial and southern sampling sites. The *C. pacifica* S morphotype is confined to the large South Pacific gyre (Figure 5).

**Figure 7:**
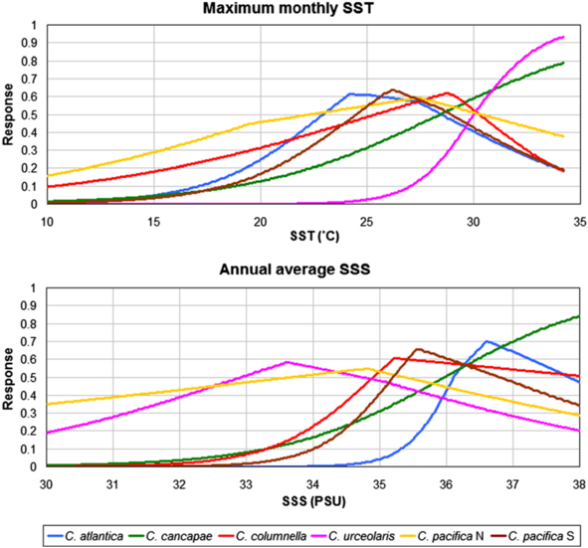
**Ecological niche modelling response curves for each** ***Cuvierina*** **morphotype.** Only response curves for maximum monthly Sea Surface Temperature (SST) and annual average Sea Surface Salinity (SSS) are shown.

Based on response curves, MaxEnt jackknife scores and Schoener’s *D* values, we found evidence for ecological differentiation among all six morphotypes (Figure 7, Table 2, Additional file 5). The relative contributions of individual oceanographic variables in explaining distribution patterns differed across morphotypes (Table 2). In the Atlantic Ocean, maximum monthly SST was most important in explaining the range of *C. cancapae* (41.9% contribution), indicative of its preference for warm waters (Figure 7). Other range-explaining variables were annual SST range (22.8%) and annual average SSS (34.6%). The distributional range of *C. atlantica*, however, was predominantly explained by annual average SSS (73.9%), and to a much smaller extent by maximum monthly SST (7.7%). The Indo-Pacific ranges of *C. urceolaris* were to a great extent explained by maximum monthly SST (86.3%), indicating a preference for warm waters (Figure 7), whereas maximum monthly PAR reaching the ocean surface (high) and chlorophyll *a* concentration (low) were important to the distribution of *C. columnella*. No single oceanographic variable was found to predominantly explain the broad range of *C. pacifica* N, but maximum monthly SST and near-surface chlorophyll *a* concentration (both 30.8%) and SSS (21.5%) contributed most. The distribution of *C. pacifica* S was predominantly defined by maximum monthly chlorophyll *a* concentration (57.1%; Table 2), indicative of a preference for waters with low surface phytoplankton biomass. All six ENMs per morphotype were accurate according to significance testing using presence-only data (AUC-values 0.884–0.9588; lowest *p* = <0.03).

**Table 2:** **MaxEnt Jackknife scores representing the relative importance of environmental variables in explaining distribution patterns of** ***Cuvierina*** **morphotypes**

**% Contribution**	***C. atlantica***	***C. cancapae***	***C. columnella***	***C. urceolaris***	***C. pacifica*** **N**	***C. pacifica*** **S**
**SST max.**	7.7	41.9	9.1	86.3	30.8	12.5
**Chlorophyll** ***a*** **max.**	3.9	0.7	53.4	3.3	30.8	57.1
**SSS average**	73.9	34.6	1.6	5.9	21.5	7.0
**PAR max.**	4.1	0.0	31.5	0.0	12.3	7.5
**SST range**	9.3	22.8	2.6	0.7	4.6	2.0
**pH average**	1.1	0.0	1.8	3.8	0.0	13.9

SST max. = maximum monthly sea surface temperature (SST), Chlorophyll *a* max. = maximum monthly near-surface chlorophyll *a* concentration (set to a maximum of 10 mg/m^3^), SSS average = annual average sea surface salinity (SSS, set to a minimum of 30 PSU), PAR max. = maximum monthly photosynthetically active radiation reaching the ocean surface (PAR), SST range = annual SST range, pH average = annual average pH. Highest Jackknife scores represent largest contributions of environmental variables to explaining geographic distributions of morphotypes, but do not provide information on values of selected environmental variables.

## Discussion

### Species boundaries

By conducting a global study combining phenotypic, genetic and biogeographic analyses of a zooplankton taxon, we show that the approach of integrative taxonomy can greatly improve our understanding of biodiversity and evolution in the open ocean. We have revealed congruent morphological, genetic and ecological patterns in *Cuvierina*, which all proved to be informative for distinguishing between taxa. We found evidence for six distinct morphotypes based on variation in shell shape and size (Figure 3, Table 1). This is one more than was formally described by [25], although he mentioned the existence of two geographic types of *C. pacifica*. We distinguished three major genetic clades: the Atlantic clade with morphotypes *C. atlantica* and *C. cancapae*, the Indo-Pacific clade with *C. columnella*, *C. urceolaris* and *C. pacifica* N, and the South Pacific clade that consisted entirely of *C. pacifica* S (Figure 4). Morphotypes *C. atlantica* and *C. cancapae* are endemic to the Atlantic, *C. pacifica* N and *C. pacifica* S are restricted to the Pacific, and *C. columnella* and *C. urceolaris* occur in both the Indian and Pacific oceans (Figure 5). All six morphotypes were clearly disjunct in terms of the combination of shell shape and size, and could be consistently distinguished by LDA. Notably, morphotypes within the same genetic clade were usually the most divergent in morphometric characters. We also found differences in oceanographic habitat preferences, with differences particularly notable within the same genetic clades and ocean basins (see Figures 5, 7 and Additional file 6). In contrast to the terrestrial domain, the application of ecological niche models for depicting ecological tolerances of pelagic taxa has been rare (e.g. [87,88]). Our ecological niche models have not taken into account diel vertical migration, dispersal limitation and biotic interactions in the prediction of the potential realized niche of the six morphotypes [88]. This may explain why morphotypes were not observed throughout their entire modelled potential niches.

Given the widely documented importance of shell morphology in the adaptation of gastropods (e.g. [89,90]), the phenotypic variation found in *Cuvierina* taxa may reflect the interplay between genetic adaptation as well as plasticity in response to environmental conditions. For example, at present there is no genetic evidence for concluding that the *C. atlantica* and *C. cancapae* morphotypes are distinct species. However, genetic differentiation may well be present in other parts of the genome and would be an interesting topic for future research. We hypothesize that interbreeding between *C. atlantica* and *C. cancapae* is unlikely given the clearly disjunct morphologies of adult shells congruent with their respective geographic distributions. The preference of *C. cancapae* for warmer waters with lower viscosities may explain its more bottle-shaped morphology with a larger surface to body weight-ratio compared to *C. atlantica*. This could be an adaptation to increase drag and thereby reduce sinking rates in warmer waters [91]. Furthermore, *C. cancapae* has pronounced shell micro-ornamentation which may also increase drag, whereas *C. atlantica* shells are completely smooth. All extinct *Cuvierina* and *Ireneia* taxa had pronounced shell micro-ornamentation and occurred in warm waters [25], suggesting that micro-ornamentation is the ancestral character state. Common garden studies of other gastropod molluscs have shown that axes of shell shape variation often have a large genetic component, such as in *Nucella lapillus* (h^2^ of 0.51, [92]) and *Littorina saxatilis* (h^2^ of 0.35–0.7, [93]).

We tentatively consider interbreeding between morphotypes within the Indo-Pacific clade unlikely as well, pending additional sampling and molecular data. We find clearly disjunct morphologies, extreme size differences and strong ecological preferences of *C. urceolaris* (warm tropical waters), *C. columnella* and *C. pacifica* N (both in subtropical, oligotrophic gyres) morphotypes. The shell of *C. urceolaris* is bottle-shaped with pronounced micro-ornamentation similar to *C. cancapae*. This may be explained by the same hypothesis as for *C. cancapae* in the Atlantic Ocean, because *C. urceolaris* also occurs in the warmest waters with lowest viscosities in the Indo-Pacific. Similar to our findings, other studies have reported much stronger phenotypic divergence than (neutral) genetic divergence in marine populations (e.g. [94]), in particular in taxa with high dispersal potential (e.g. [21,95-97]).

Ecological barriers to dispersal and similar distribution patterns as for *Cuvierina* are found in several other open ocean plankton taxa, such as *Diacavolinia* pteropods, foraminifers, krill and copepods. *Diacavolinia flexipes, D. angulosa* and *D. grayi* show a zonation of equatorial and subtropical distribution patterns in the Indian Ocean similar to *C. urceolaris* and *C. columnella* distributions [24]. Like *C. pacifica* S, the krill species *Euphausia gibba* is endemic to the large South-Pacific gyre [98]. The presence of a disjunct distribution pattern as for *C. atlantica* in the Atlantic Ocean is also observed for the pelagic copepod genus *Pleuromamma* [99,100] and the mesopelagic copepod *Haloptilus longicornis* [101]. However, unlike *H. longicornis*, the *C. atlantica* morphotype is unable to reach the Indian Ocean around South Africa, where a warm current from the Indian Ocean impinges a cold current from the Southern Ocean. Numerous clades of planktonic foraminifers also have distribution patterns that are predominantly based on latitudinal zonation of water masses (e.g. [17]).

Based on results from this study, we do not find support for a subdivision into the subgenera *Cuvierina* (containing *C. atlantica*, *C. columnella*, *C. pacifica* N and *C. pacifica* S) and the more bottle-shaped *Urceolarica* (*C. urceolaris* and *C. cancapae*) as proposed by [25,38]. We find evidence that *C. pacifica* N and *C. pacifica* S are distinct species because they belong to different genetic clades (COI and 28S) and are morphologically disjunct. Because the holotype of *C. pacifica* belongs to the *C. pacifica* S morphotype, we propose a limitation of the description of *C. pacifica* to *C. pacifica* S and a new taxonomic description for the *C. pacifica* N morphotype (Burridge et al., in prep.). However, because we found no molecular evidence to support the species status of morphotypes within the Atlantic and Indo-Pacific clades, we consider it possible that they represent ecophenotypic varieties or incipient species.

### Evolution in *Cuvierina*

Based on a fossil-calibrated molecular phylogeny, we propose a biogeographic scenario for the evolution of *Cuvierina* morphotypes that is influenced by decreasing connectivity between the three world oceans since the Miocene (Figure 6). The now extinct ancestral genera *Spoelia*, *Johnjagtia* and *Ireneia* originated during the Oligocene at 34–33 mya as an offshoot of the Creseidae according to [25]. The first *Cuvierina* was thought to have originated directly from *Ireneia* [25]. However, the outgroup relationships of *Cuvierina* remain poorly resolved possibly because ancestral genera, as well as many Miocene and Pliocene *Cuvierina* taxa, are now extinct (e.g. *C. torpedo*, *C. paronai*, *C. grandis*, *C. curryi*, *C. intermedia*, *C. jagti*, *C. inflata*, *C. ludbrooki*, *C. astesana*, and *C. miyakiensis*; [25,38,102-104]). There was high connectivity between the three world oceans at the time of divergence between the South Pacific genetic clade and the Atlantic and Indo-Pacific *Cuvierina* clades at 25.3 (28–23) mya (Late Oligocene), but it is unknown for how long the South Pacific clade has been endemic to the large South Pacific gyre. The divergence between the Atlantic and Indo-Pacific clades of 16.1 (24.5–7) mya (Miocene) coincides with the Tethys Sea closure, suggesting a vicariant model of divergence, with the Indo-Pacific clade diverging from the Atlantic clade in the Indian Ocean and later dispersing to the Pacific basin. Until the Terminal Tethyan Event (TTE, 12–18 mya, [86]), the Atlantic and Indian oceans were connected through the Tethyan Seaway, but pelagic connectivity was probably reduced from ± 21 mya onwards [105,106]. Dispersal between the Atlantic and Pacific oceans was possible until ± 3.1 mya with the final closure of the Isthmus of Panama (IOP, [86]). However, there are no clear indications of vicariance events of Atlantic and Pacific *Cuvierina* associated with the IOP (Figure 6). The estimated TMRCAs of the three extant clades correspond very nicely with the estimates based on fossil evidence (5 – 4 mya; [25]).

Connectivity between the three major oceans is now much more restricted at tropical and subtropical latitudes than in the Early Miocene. The Indo-West-Pacific region (IWP) does not seem to represent a physical barrier today, but could have functioned as such between Indian and Pacific populations of *C. columnella* and *C. urceolaris* during glacial periods when sea levels dropped (e.g. [107]). This is a possible explanation for the subtle morphometric differences between Indian and Pacific *C. urceolaris* and *C. columnella* specimens. The IWP has also been considered an intermittent barrier to dispersal for several copepods [99,108]. In these studies, significant population genetic structuring was found between Indian and Pacific populations of *Eucalanus hyalinus* and *E. spinifer* [108] and *Pleuromamma xiphias* [99].

Physical and ecosystem properties of different Atlantic water masses may be key to incipient speciation within the Atlantic clade. Our population genetic analyses of *C. atlantica* suggest that there is a significant degree of structuring between populations in the northern and southern subtropical gyre systems in the Atlantic Ocean. If genetic structure is interpreted in light of gene flow, this implies a more limited dispersal than expected for open ocean holoplankton. Combined with the disjunct distribution of *C. atlantica* populations, this suggests that the equatorial upwelling waters in the Atlantic represent a significant barrier to dispersal. This equatorial dispersal barrier was also found for the mesopelagic copepod *Haloptilus longicornis*, for which a genetic break was observed between populations in the northern and southern oligotrophic Atlantic gyres [101]. By contrast, the equatorial Atlantic offers an ecological niche for *C. cancapae*.

## Conclusion

Given the distinct ecological and phenotypic specializations found among both described and undescribed *Cuvierina* taxa, they may not respond equally to future ocean changes and may not be equally sensitive to ocean acidification. Because the presence and strength of open ocean dispersal barriers depends on the ecological niche of a species, the capacity of a species to adapt to or to track a suitable habitat may vary across closely-related taxa [13]. Although open ocean evolution is partially driven by vicariance, our findings support the view that ecological differentiation may also be a major driving force of speciation for zooplankton.

## Availability of supporting data

The data set supporting the results of this article is available at the Dryad repository, [109], and DNA sequences have been deposited at GenBank under the following accession numbers: KP292620 – KP292794.

## Additional files

Additional file 1:
**Extended summary of** ***Cuvierina*** **samples used in this study.**
Additional file 2:
**Ordination of uncorrected RW data of** ***Cuvierina*** **in an apertural orientation.** Fresh (N = 115), reference museum (N = 352) and other museum specimens (N = 83) are included. Relative Warp 1 explains 56.01% of the total shape variation; RW2 explains 22.86%. Corresponding thin plate splines of the most positive and negative deformations along the axes are indicated to depict the variation in shell shape. Six distinguished morphotypes are indicated in the legend. Additional file 3:
**Maximum likelihood tree of 30** ***Cuvierina*** **specimens and 6 outgroup taxa using COI (658 bp) and 28S (989 bp).** Numbers indicate bootstrap support (only bootstrap values of major clades are shown). Symbols for *Cuvierina* indicate major genetic clades; colours indicate distinct morphotypes (also see Figure 5). Additional file 4:
**Pairwise genetic distances within and between** ***Cuvierina*** **morphotypes based on COI and 28S.**
Additional file 5:
**Assessment of ecological niche overlap between** ***Cuvierina*** **morphotypes using Schoener’s** ***D*** **.**
Additional file 6:
**Geographic overview of all** ***Cuvierina*** **specimens used in this study.** Sampling locations are projected on a map of annual average sea surface salinities (SSS) (MARSPEC data set, [84]). See legend for explanation of symbols and colours.
