# Supplementary figures and images for: Global biogeography and evolution of Cuvierina pteropods
Source: BMC Evol Biol. 2015 Mar 12;15:39. doi: 10.1186/s12862-015-0310-8 (PMC4443520; doi:10.1186/s12862-015-0310-8)

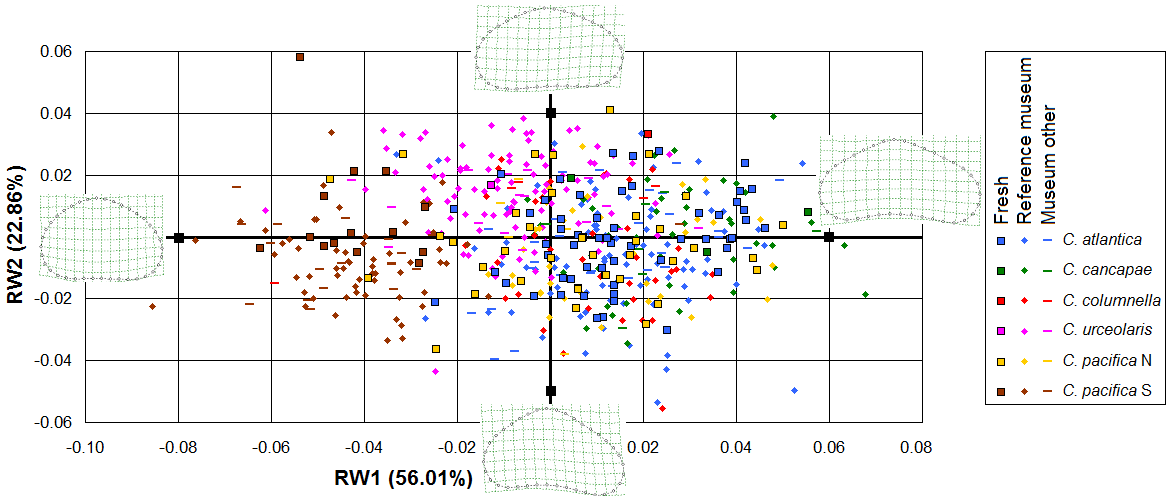

Supplement: Additional file 2: — Ordination of uncorrected RW data of Cuvierina in an apertural orientation. Fresh (N = 115), reference museum (N = 352) and other museum specimens (N = 83) are included. Relative Warp 1 explains 56.01% of the total shape variation; RW2 explains 22.86%. Corresponding thin plate splines of the most positive and negative deformations along the axes are indicated to depict the variation in shell shape. Six distinguished morphotypes are indicated in the legend. [file 12862_2015_310_MOESM2_ESM.png]

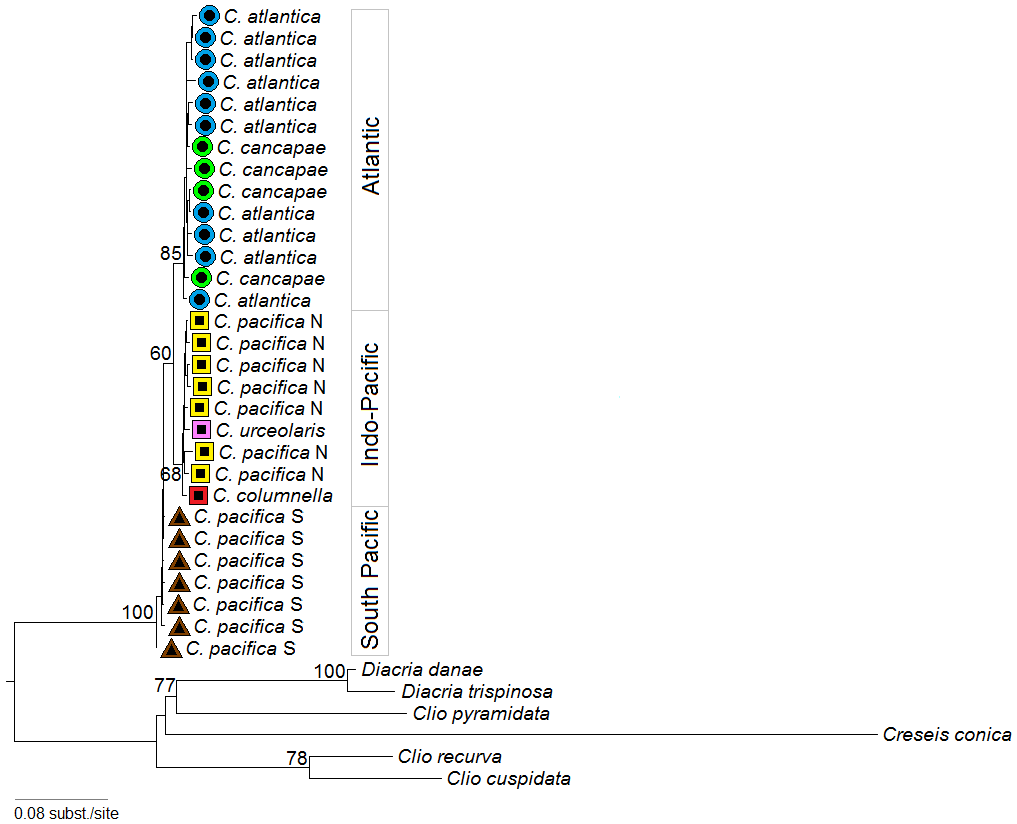

Supplement: Additional file 3: — Maximum likelihood tree of 30 Cuvierina specimens and 6 outgroup taxa using COI (658 bp) and 28S (989 bp). Numbers indicate bootstrap support (only bootstrap values of major clades are shown). Symbols for Cuvierina indicate major genetic clades; colours indicate distinct morphotypes (also see Figure 5). [file 12862_2015_310_MOESM3_ESM.png]

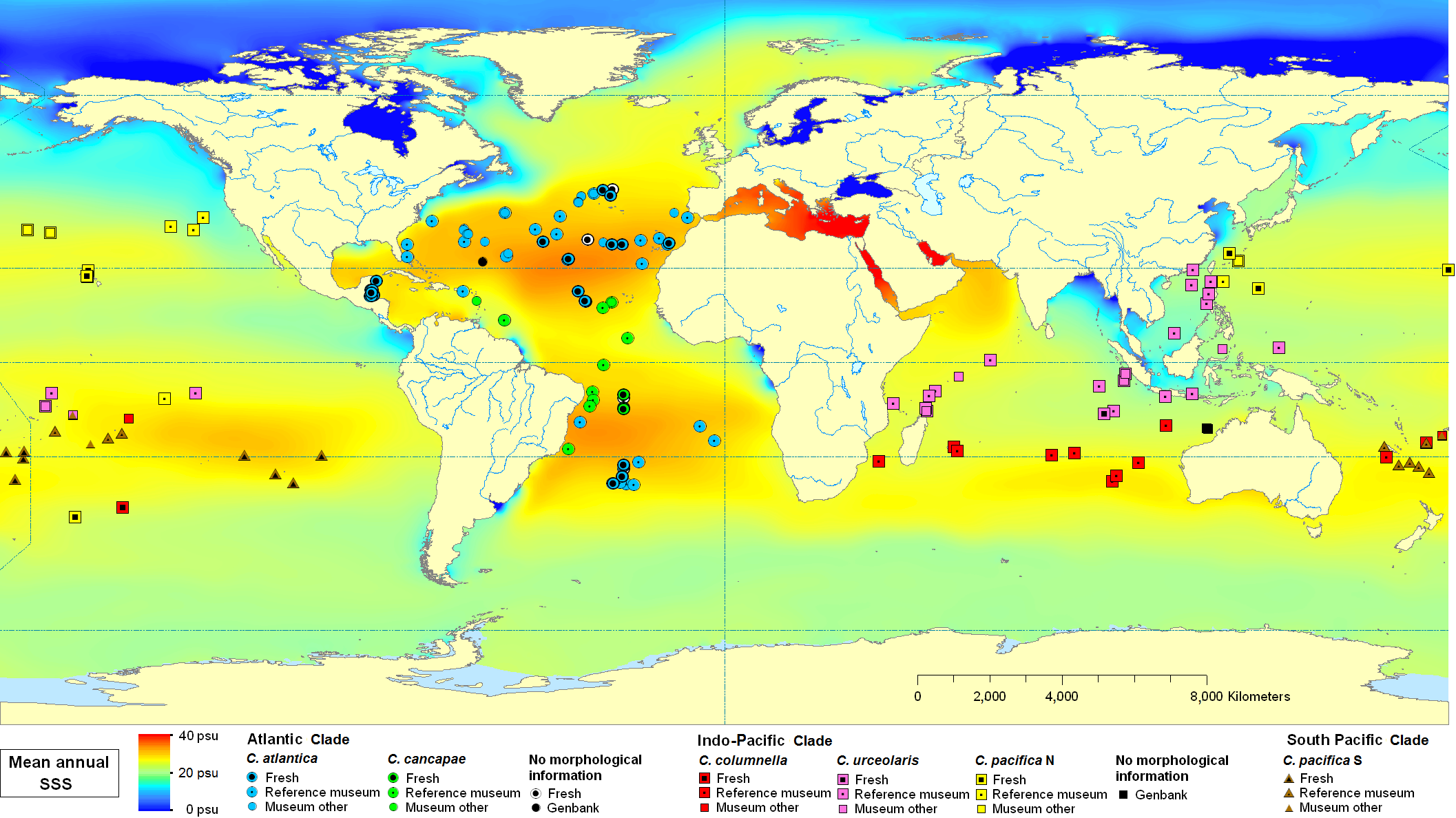

Supplement: Additional file 6: — Geographic overview of all Cuvierina specimens used in this study. Sampling locations are projected on a map of annual average sea surface salinities (SSS) (MARSPEC data set, [84]). See legend for explanation of symbols and colours. [file 12862_2015_310_MOESM6_ESM.png]
